# Developmental dynamics and functional adaptation of gut microbiota in Mongolian wild asses (*Equus hemionus hemionus*) across ontogenetic stages in arid desert ecosystems

**DOI:** 10.3389/fmicb.2025.1659661

**Published:** 2025-09-17

**Authors:** Jianeng Wang, Haifeng Gu, Hongmei Gao, Tongzuo Zhang, Bin Li, Meng Zhang, Feng Jiang, Pengfei Song, Chengbo Liang, Qing Fan, Youjie Xu, Ruidong Zhang

**Affiliations:** ^1^Key Laboratory of Adaptation and Evolution of Plateau Biota, Northwest Institute of Plateau Biology, Chinese Academy of Sciences, Xining, China; ^2^College of Life Sciences, University of Chinese Academy of Sciences, Beijing, China; ^3^Inner Mongolia Normal University, Hohhot, China; ^4^Qinghai Provincial Key Laboratory of Animal Ecological Genomics, Xining, China; ^5^Urad Middle Banner Management Station of National Nature Reserve of Haloxylon Ammodendron and Equus Hemionus, Bayannur, China; ^6^Urad Rear Banner Management Station of National Nature Reserve of Haloxylon Ammodendron and Equus Hemionus, Bayannur, China

**Keywords:** developmental stages, gut microbiota, metagenomics, Mongolian wild ass, arid ecosystem adaptation, functional annotation

## Abstract

Understanding the composition and function of gut microbiota is essential for elucidating how wild animals adapt to arid environments. The Mongolian wild ass (*Equus hemionus hemionus*), which inhabits harsh desert ecosystems, offers an ideal model for such investigations. This study employed metagenomic sequencing of fecal samples to characterize the composition and structure of the gut microbiota in adult, subadult, and juvenile Mongolian wild asses, with functional annotation based on the KEGG, CARD, and CAZy databases. Our study revealed that Bacillota and Bacteroidota were the dominant phyla, together accounting for over 85% of relative abundance, with their ratio (B/B value) showing clear age-dependent shifts. Juveniles were dominated by Bacillota (high B/B value), consistent with adaptation to a milk-based, protein- and lactose-rich diet, whereas adults shifted toward Bacteroidota dominance (low B/B value), with relative abundance increasing from 39.53% to 64.92%, reflecting enhanced polysaccharide and fiber degradation, thereby providing microecological support for adaptation to low-energy, high-fiber desert vegetation resources. Alongside this transition, α-diversity significantly increased with age, while β-diversity patterns shifted from dispersed to clustered, indicating a more complex, stable, and mature gut community. Over 58% of predicted genes were assigned to metabolic pathways, highlighting the essential contribution of gut microbes to herbivore digestion. Polysaccharide lyases, enriched in adults and subadults, were positively correlated with Bacteroidota abundance, highlighting their central role in fiber degradation and stable energy supply, thereby supporting adaptation to arid desert habitats. In contrast, juveniles were characterized by enrichment of the galactose metabolism pathway, high abundance of Pseudomonadota (LEfSe LDA > 4), and the largest number of antibiotic resistance genes (AROs), including 17 potential key AROs, reflecting greater microbial plasticity and higher environmental exposure risks during early development. This study provides the first systematic characterization of age-related gut microbiome dynamics and functional adaptations in the endangered Mongolian wild ass, offering novel insights into microbial contributions to host energy optimization and resilience in arid ecosystems, with implications for conservation strategies.

## 1 Introduction

The impact of global climate change on wildlife populations has become increasingly pronounced (Bellard et al., 2012). Among its many consequences, the rising frequency and intensity of drought events stands out as one of the most direct and ecologically far-reaching outcomes (Perkins-Kirkpatrick and Lewis, 2020). Arid environments are among the most challenging ecosystems on Earth, characterized by low precipitation, high evaporation rates, sparse vegetation cover, large diurnal temperature fluctuations, and extreme resource scarcity (Huang et al., 2017). Wildlife inhabiting these regions are exposed to multiple survival pressures, including dehydration, food shortages, and thermal stress (Fuller et al., 2021). Changes in temperature and precipitation driven by climate change have been shown to significantly affect wildlife survival and reproductive success (Paniw et al., 2019; Groenewoud and Clutton-Brock, 2021). To cope with these extreme conditions, various species have evolved a range of physiological, behavioral, and ecological strategies, such as efficient water retention mechanisms, seasonal migration, and flexible metabolic regulation (Fick et al., 2009; Mitchell et al., 2018; Levy et al., 2019; Weyer et al., 2020).

The Mongolian wild ass (*Equus hemionus hemionus*), a subspecies of the Asiatic wild ass (*Equus hemionus*) within the genus *Equus* and family Equidae, is a representative species of Central Asia’s arid desert regions. Historically, it was widely distributed across typical steppe and desert-steppe habitats at elevations ranging from 800 to 2,500 meters in central Asia (Bi et al., 2007; Zhang et al., 2020). In recent years, however, both its population size and range have declined due to the combined pressures of climate change and human disturbance. Despite this, the species continues to play an irreplaceable ecological role in maintaining the structural stability and functional continuity of arid desert-steppe ecosystems (Gao et al., 2024). Owing to its long-term habitation in extremely dry and resource-scarce environments, the Mongolian wild ass is believed to possess exceptional environmental adaptability and has long been recognized as an important model species for studying adaptive mechanisms in desert ecosystems. Therefore, a comprehensive understanding of its physiological and ecological adaptation strategies under arid environments is of great scientific and conservation significance.

In recent years, research has revealed that the host’s own physiological and behavioral adjustments may not fully account for the long-term survival advantages observed in certain species inhabiting extreme environments (Boothby, 2019). The gut microbiota, acting as a critical interface between the host and its environment, plays a vital role in maintaining physiological homeostasis, modulating thermal tolerance, and defending against pathogens (Honda and Littman, 2016; Risely et al., 2023). For example, some symbiotic bacteria can enhance host thermal resilience or suppress pathogens through immune modulation and direct competition, thereby improving host survival under adverse conditions (Belkaid and Hand, 2014; Harris et al., 2019; Moeller et al., 2020). Studies suggest that under new environmental pressures, protective microbial strains or functional groups may become enriched within the host as a result of selection, either through direct filtering of beneficial microbes or through host-mediated changes in behavior or development that facilitate their colonization and transmission (Moeller and Sanders, 2020). However, research on the gut microbiota of Mongolian wild asses remains scarce. Available studies suggest that wild individuals harbor greater microbial diversity and richness than captive ones, likely reflecting adaptation to the nutrient-poor Gobi Desert (Jarquín-Díaz et al., 2025). Comparative analyses further indicate that Mongolian wild asses exhibit distinctive diversity patterns compared with Przewalski’s horses (*Equus przewalskii*) and domestic horses (*Equus caballus*), implying a unique gut microbial community structure (Hu et al., 2022). These limited findings suggest that Mongolian wild asses may possess unique gut microbial structures and ecological features. Therefore, elucidating the composition and functional traits of their gut microbiota represents a key step toward uncovering the mechanisms underlying their adaptation to arid environments.

Although significant progress has been made in wildlife ecological research on gut microbiota, current studies have primarily focused on adult individuals, often overlooking the dynamic shifts in microbial communities across different ontogenetic stages. Ontogenetic evidence shows that host-associated gut microbial diversity and function can vary substantially during development (Turnbaugh et al., 2007; Mariat et al., 2009; Badal et al., 2020). This has been demonstrated in species such as the western lowland gorilla (*Gorilla gorilla gorilla*) (Pafčo et al., 2019), chimpanzee (*Pan troglodytes*) (Reese et al., 2021), chinstrap penguin (*Pygoscelis antarctica*) (Barbosa et al., 2016), Tibetan sheep (*Ovis aries*) (Wang et al., 2019), sika deer (*Cervus nippon*) (Li et al., 2019), and Bactrian camel (*Camelus bactrianus*) (He et al., 2019). Consequently, neglecting microbial dynamics across ontogenetic stages may limit our systemic understanding of life cycle-driven adaptive strategies in animals, and findings may be confounded by “averaging effects” that obscure critical host-microbe interactions during key physiological transitions such as weaning. Therefore, adopting a life-cycle perspective to systematically examine the coordination between gut microbial composition and function across developmental stages in the Mongolian wild ass will not only help elucidate the mechanisms underpinning micro-ecosystem dynamics during host growth, but also provide a theoretical basis for understanding adaptive strategies shaped by life-history processes under arid conditions, thereby informing age-specific conservation and management strategies for wild populations.

## 2 Materials and methods

### 2.1 Sample collection and preservation

In November 2024, fresh fecal samples of Mongolian wild asses were collected using a non-invasive sampling approach in a desert-steppe habitat in Urad Middle Banner, Inner Mongolia Autonomous Region, China. To minimize the risk of cross-contamination by environmental DNA, sampling was conducted in areas free from precipitation or strong winds (≥ 10 m/s) within the previous 24 h. Prior to sample collection, binoculars were used to observe the behavior of wild asses, and feces were collected immediately after defecation was observed.

Fresh feces were identified based on the following criteria: a dark brownish-green and glossy exterior, a grass-green moist interior, and a soft texture. These standards ensured the collection of high-quality samples. The developmental stage of each individual was inferred based on fecal size, as significant differences in volume and diameter allow for reliable visual distinction among adults, subadults, and juveniles (Supplementary Figure 1). In addition, deposition morphology provided further discriminatory power: adult feces, being heavier, typically occurred as discrete pellets, juvenile feces, being lighter, were more often clustered or strip-like, and subadult feces exhibited intermediate features. These morphological criteria can serve as auxiliary validation for age-class determination. A total of 21 fecal samples were collected, ensuring individual independence through spatial separation (≥ 100 meters apart) and visual confirmation of distinct defecation events.

Approximately 5 grams of fecal material were collected from the interior of each sample—avoiding any part in contact with the ground—using disposable sterile gloves. The samples were immediately transferred to sterile cryogenic tubes, flash-frozen in liquid nitrogen, and subsequently stored at −80 °C to minimize degradation. This preservation protocol follows established standards in microbiome research and has been validated for its effectiveness.

### 2.2 Sex determination and sample grouping

To eliminate the potential confounding effects of sex on experimental outcomes, all collected fecal samples underwent genetic sex determination. DNA was extracted from each fecal sample using the HiPure Universal DNA Kit (Genepioneer, Nanjing, China), and PCR amplification was conducted in a 20 μL reaction volume, comprising: 10 μL of 2 × Taq PCR MasterMix, 1 μL each of forward and reverse primers (10 μM), 1 μL of DNA template, and ddH2O added to a final volume of 20 μL. The primers used were: forward 5′-GCAATTCATTAGTATCCGTCTGTG-3′ and reverse 5′-AATCAAGGAAAATCACCGTGAGA-3′. The PCR program included an initial denaturation at 95 °C for 5 min, followed by 30 cycles of 95 °C for 30 s, 58 °C for 30 s, and 72 °C for 1 min, with a final extension at 72 °C for 10 min. PCR products were separated on 1.5% agarose gels, stained with GelRed, and visualized under ultraviolet light. A DL2000 DNA Marker (100–2,000 bp; Tsingke Biotech, Beijing, China; Cat. No. TSJ011-100) was used as a molecular weight reference. After determining the sex of each individual based on the banding patterns, only female samples were retained for subsequent analyses. Two male samples were excluded, resulting in a final dataset of 19 female samples. These were categorized into three developmental stages: adult group (AD, *n* = 8; samples AD11-1 to AD11-8), subadult group (SA, *n* = 6; samples SA11-1 to SA11-6), and juvenile group (JU, *n* = 5; samples JU11-1 to JU11-5).

### 2.3 Metagenomic sequencing and annotation

Total DNA was re-extracted from fecal samples using the HiPure Universal DNA Kit (Genepioneer, Nanjing, China). DNA integrity was assessed via 1% agarose gel electrophoresis, while DNA concentration was measured using a Qubit fluorometer and purity was evaluated with a Nanodrop spectrophotometer. Extracted DNA was then sheared to an average fragment size of 300 bp using a Covaris M220 ultrasonicator. Paired-end (PE) libraries were constructed using the TruSeq™ DNA Sample Prep Kit and enriched by PCR, followed by PE150 sequencing on the Illumina NovaSeq 6000 platform (Q30 ≥ 90%). Raw sequencing reads were quality-filtered using fastp (v0.20.0, parameters: -q 5 -n 5). High-quality reads were then mapped to the Mongolian wild ass reference genome (NCBI Assembly ID: GCF_041296235.1) using Bowtie2 (v2.3.5.1) to remove host-derived sequences. On average, each sample yielded approximately 13.04 Gb of high-quality data. Clean reads were individually assembled using MEGAHIT (v1.2.9, parameters: −min-contig-len 500, −presets meta-large, Li et al., 2015). Open reading frames (ORFs) were predicted using Prodigal (v2.6.3, parameters: -p meta -m, Noguchi et al., 2006; Hyatt et al., 2010). Predicted ORFs were clustered with CD-HIT (v4.8.1, parameters: -c 0.95 -aS 0.9 -g 1 -r 1 -d 0, Li and Godzik, 2006) to generate a non-redundant gene catalog (Unigenes). Clean reads from each sample were then aligned to the Unigenes using BWA [v0.7.71, (Li and Durbin, 2009)] to calculate gene abundance, and genes with fewer than two aligned reads across all samples were filtered out, resulting in the final Unigene dataset.

For taxonomic annotation, Kraken [v2.1.2 (Wood and Salzberg, 2014)] was employed to classify Unigenes based on k-mer exact matching using the default parameters. The database included reference genomes from Bacteria, Fungi, Archaea, and Viruses. Taxonomic profiles and gene counts were summarized at various taxonomic levels (domain, phylum, class, order, family, genus, species) based on annotation results and gene abundance. Functional annotation was conducted using multiple databases. The Carbohydrate-Active enZYmes Database (CAZy) was queried via BLAST (v2.2.26, parameters: -p blastp, e-value ≤ 1e-5). For antibiotic resistance gene (ARG) profiling, the Comprehensive Antibiotic Resistance Database (CARD) was accessed using the RGI (v5.1.0) with its built-in blastp algorithm (e-value threshold = 1e-30). Relative abundance of antibiotic resistance ontologies (AROs) was calculated based on RGI results and gene abundances. Kyoto Encyclopedia of Genes and Genomes (KEGG) functional annotation was performed using DIAMOND (v2.0.6) for alignment against the KEGG database (blastp, e-value ≤ 1e-5), generating KEGG Orthology (KO)-level relative abundance profiles and functional gene counts across all samples.

### 2.4 Data analysis

All data analyses in this study were conducted within the R software environment (version 4.3.2). Initially, the Kruskal–Wallis rank sum test was applied to compare differences in microbial composition among the three groups at the phylum, genus, and species levels. The Benjamini–Hochberg (BH) method was used to correct for multiple testing and control the false discovery rate (FDR). For microbial taxa showing significant differences, pairwise group comparisons were further conducted using the Wilcoxon rank sum test with a significance threshold of *P* < 0.05, followed by additional *p*-value adjustments. Linear Discriminant Analysis Effect Size (LEfSe) was employed to evaluate the contribution of differentially abundant taxa to group differences. Additionally, α-diversity and β-diversity indices of the gut microbiota were calculated using the vegan package. α-diversity metrics included Observed_species, Chao1, Shannon, Simpson, and PD_whole_tree indices, with group differences statistically tested. β-diversity was assessed by calculating the Bray-Curtis distance matrix using the vegan package, followed by analysis of similarities (ANOSIM) with 999 permutations to evaluate intergroup differences, and visualized via non-metric multidimensional scaling (NMDS).

Differential analysis of KO abundances obtained from alignment was performed using the Functional Mapping and Analysis Pipeline (FMAP, v0.13) with a significance cutoff of *p* < 0.05. Significantly different KOs were mapped to KEGG level 3 pathways for functional enrichment trend analysis. For antibiotic resistance genes (Antibiotic Resistance Ontologies, AROs), the SPEC-OCCU method was used to calculate specificity and occupancy of each ARO across different age groups. Specificity was defined as the proportion of the average abundance of an ARO within a group relative to the total abundance across all groups, while occupancy referred to the relative frequency of occurrence of that ARO within the group’s samples (Dufrêne and Legendre, 1997; Gweon et al., 2021). SPEC-OCCU metrics were computed using custom R scripts without relying on dedicated R packages. For CAZy family abundances showing significant differences among groups (*p* < 0.05, Kruskal–Wallis test), Mantel tests were conducted to analyze correlations between CAZy abundance and microbial community structures at phylum, genus, and species levels. Correlation analyses were performed using the mantel function from the vegan package. Visualization of all three analyses was carried out with the ggplot2 package.

## 3 Results

### 3.1 Metagenomic sequencing and gene prediction results

**FIGURE 1:**
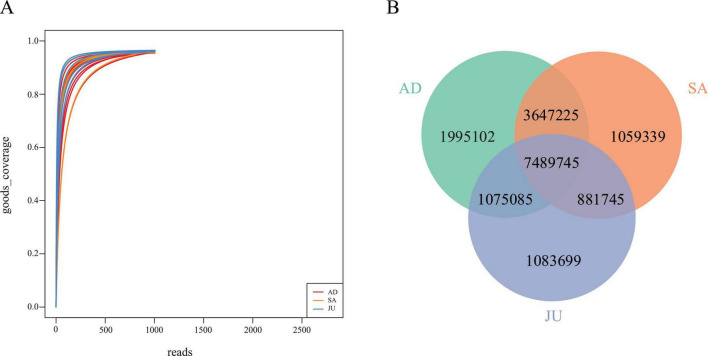
Sequencing depth and gene distribution among age groups. **(A)** Rarefaction curves of Good’s coverage for each sample. The curves plateaued near 1.0, indicating sufficient sequencing depth and comprehensive coverage of microbial diversity. **(B)** Venn diagram showing shared and unique non-redundant genes among the AD, SA, and JU. Numbers represent the total number of non-redundant genes in each intersection.

This study generated a total of 248,432.09 Mbp of raw metagenomic sequencing data from 19 Mongolian wild ass fecal samples, averaging 13,075.37 Mbp per sample. After quality control, 247,748.55 Mbp of clean data (99.72%) were retained, with an average of 13,039.40 Mbp per sample. A total of 38,089,381 coding sequences (CDS) were predicted across the 19 samples. After redundancy removal, 17,231,940 non-redundant genes remained, with an average sequence length of 626.12 bp. Good’s coverage curves showed that coverage rapidly approached 100% as sequencing depth increased, stabilizing around 1,000–1,500 reads, indicating that the sequencing depth was sufficient to capture the majority of detectable microbial species and had reached saturation (Figure 1A).

The Venn diagram (Figure 1B) revealed that AD contained 14,207,157 genes, averaging about 1,775,895 genes per sample; SA had 13,078,054 genes, averaging approximately 2,179,676 genes per sample; and JU contained 10,530,274 genes, averaging around 2,106,055 genes per sample. Across the three groups, 7,489,745 genes were shared, accounting for 43.46% of the total genes. Specifically, the shared gene proportion between the AD and SA was 68.97%, between AD and JU was 52.96%, and between SA and JU was 54.94%.

### 3.2 Gut microbiota composition and α-diversity

**FIGURE 2:**
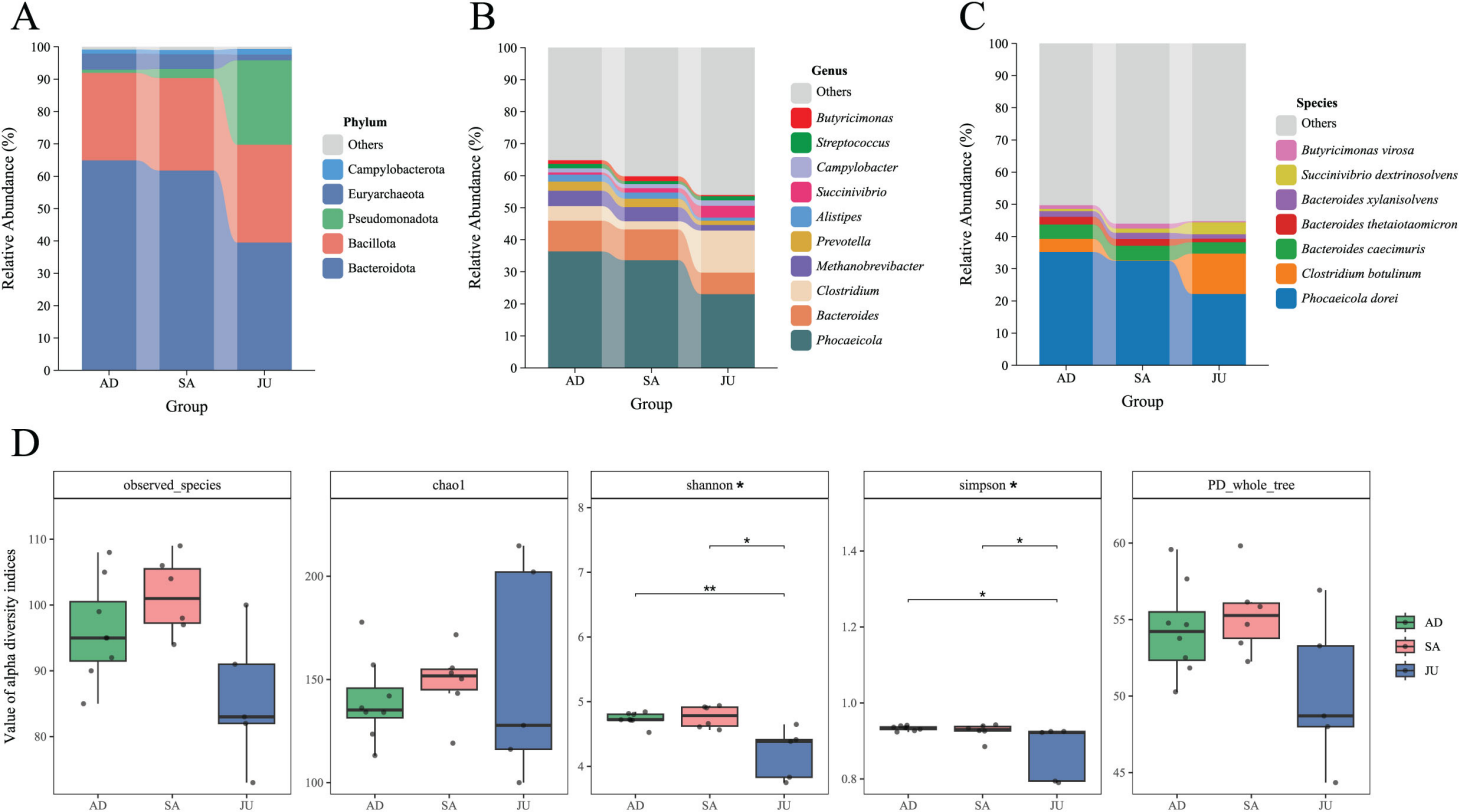
Gut microbiota composition and α-diversity across developmental stages. **(A)** Relative abundance of dominant bacterial phyla (> 1%) in the AD, SA, and JU. **(B)** Relative abundance of dominant bacterial genera (> 1%) in each group. **(C)** Relative abundance of dominant bacterial species (> 1%) across groups. **(D)** α-diversity indices (Observed species, Chao1, Shannon, Simpson, PD whole tree) of gut microbiota. Box plots represent distribution per group. * indicates *p* < 0.05, ** indicates *p* < 0.01 (Wilcoxon test with FDR correction).

Based on metagenomic sequencing data, this study analyzed gut microbial community annotations at the phylum, genus, and species levels for the three groups, calculating the relative abundance of each taxon. Dominant taxa with relative abundance greater than 1% were selected, and stacked bar plots were generated to illustrate their abundance distribution across groups (Figures 2A–C). At the phylum level, the main dominant phyla were Bacteroidota (57.25%), Bacillota (28.37%, formerly Firmicutes), Pseudomonadota (8.15%), Euryarchaeota (4.00%), and Campylobacterota (1.40%). Comparisons among groups showed a decreasing trend of Bacteroidota from AD (64.92%), SA (61.78%), to JU (39.53%). In contrast, Bacillota abundance remained relatively stable with a slight increase across groups: AD (27.05%), SA (28.60%), and JU (30.21%). Pseudomonadota was notably higher in the JU (26.07%) compared to AD (0.95%) and SA (2.80%), showing an increasing trend among the three groups. Euryarchaeota abundance was lowest in the JU (1.79%) and higher in AD (4.96%) and SA (4.56%), showing a decreasing trend. Campylobacterota abundance was stable among the groups: AD (1.29%), SA (1.29%), and JU (1.73%). At the genus level, the dominant genera in the AD included *Phocaeicola* (36.38%), *Bacteroides* (9.61%), *Clostridium* (4.56%), *Methanobrevibacter* (4.80%), *Prevotella* (2.83%), and *Alistipes* (2.18%). The SA was dominated by *Phocaeicola* (33.66%), *Bacteroides* (9.59%), *Methanobrevibacter* (4.38%), *Prevotella* (2.66%), and *Clostridium* (2.57%). The JU was mainly represented by *Phocaeicola* (23.03%), *Clostridium* (13.13%), *Bacteroides* (6.75%), and *Succinivibrio* (3.68%). The abundance of *Phocaeicola*, *Bacteroides*, *Methanobrevibacter*, *Prevotella*, and *Alistipes* decreased progressively from AD through SA to JU. At the species level, seven species with relative abundance greater than 1% were identified: *Phocaeicola dorei* (30.92%), *Clostridium botulinum* (5.06%), *Bacteroides caecimuris* (4.25%), *Bacteroides thetaiotaomicron* (1.95%), *Bacteroides xylanisolvens* (1.72%), *Succinivibrio dextrinosolvens* (1.68%), and *Butyricimonas virosa* (1.06%).

To comprehensively assess α-diversity of gut microbiota across age groups, five common α-diversity indices were calculated at the species level (Supplementary Table 1), including species richness indices Observed_species and Chao1, diversity indices Shannon and Simpson, and phylogeny-based PD_whole_tree. The results indicated differences in α-diversity metrics among groups. Kruskal–Wallis rank sum tests revealed significant differences in Shannon and Simpson indices (*p* = 0.0474). Subsequent pairwise Wilcoxon rank sum tests with FDR correction revealed that the Shannon index was significantly higher in AD (*p* = 0.0093) and SA (*p* = 0.0260) than in JU. Similarly, the Simpson index was elevated in both AD (*p* = 0.0186) and SA (*p* = 0.0455) compared with JU (Figure 2D).

### 3.3 Intergroup differences analysis

**FIGURE 3:**
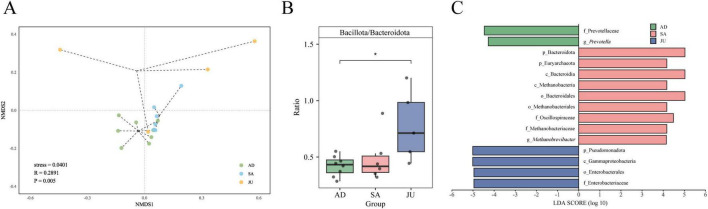
β-diversity and group-specific microbial markers. **(A)** NMDS plot based on Bray–Curtis dissimilarity showing gut microbiota structure at the species level. Each point represents one sample colored by group. Stress = 0.0401; ANOSIM *R* = 0.2891, *p* = 0.005. **(B)** Bacillota to Bacteroidota (B/B) ratio comparison across the AD, SA, and JU. * indicates *p* < 0.05. **(C)** Differential microbial taxa identified by LEfSe analysis (LDA score > 4).

To further assess differences in gut microbial community structure among groups, this study performed NMDS based on Bray–Curtis distance matrices. The visualization showed a certain degree of separation of samples in the two-dimensional coordinate system (stress = 0.0401, Figure 3A). However, the JU exhibited greater within-group variation, suggesting that the gut microbiota in juvenile donkeys is still undergoing establishment and dynamic adjustment, and the individual microbiome structures have not yet stabilized. Subsequently, ANOSIM analysis was conducted to statistically test intergroup differences. The results showed that differences between groups were significantly greater than within groups (*R* = 0.2891, *p* = 0.005), further confirming the statistically significant differences in microbial community composition among groups.

To identify significant differences in microbial composition among the groups, non-parametric tests with FDR correction were applied. The results are summarized in Supplementary Table 2, which presents the mean, standard deviation, and statistical outcomes for phyla and genera with relative abundance greater than 1% across the three groups. At the phylum level, Pseudomonadota abundance was significantly higher in the JU compared to the AD (*p* = 0.0047) and SA (*p* = 0.0455). Euryarchaeota abundance was significantly higher in the AD compared to the SA (*p* = 0.0047) and JU (*p* = 0.0065). Moreover, the Bacillota/Bacteroidota (B/B) ratio in the JU was significantly higher than in the AD (*p* = 0.0186, Figure 3B). At the genus level, *Methanobrevibacter*, *Prevotella*, *Alistipes*, and *Butyricimonas* showed significantly higher abundance in both AD and SA compared to the JU. At the species level, *Butyricimonas virosa* abundance was significantly higher in the AD (*p* = 0.0047) and SA (*p* = 0.0065) than in the JU. Notably, no statistically significant differences were found between the AD and SA at these phylum, genus, or species levels.

To further identify representative gut microbial taxa in each group, LEfSe analysis was performed on the significantly different taxa for biomarker screening. Using an LDA score threshold > 4, 15 biomarkers with significant statistical differences and high impact among groups were identified (Figure 3C). Among them, taxa related to Prevotellaceae contributed most to the AD; taxa related to Bacteroidota and Euryarchaeota had higher influence in the SA; and taxa associated with Pseudomonadota and Enterobacterales were more influential in the JU.

### 3.4 Functional profiling of the gut microbiome

#### 3.4.1 KEGG analysis

To investigate the functional differences of gut microbiomes in Mongolian wild asses at different ontogenetic stages, we annotated the non-redundant genes obtained from metagenomic sequencing using the KEGG database. The results showed that a total of 6,614,234 UniGenes (accounting for 38.38% of the total) were successfully annotated and assigned to 5,311 KOs. At KEGG Level 1 classification, six functional categories were identified, with “Metabolism” exhibiting the highest relative abundance (58.03% ± 0.23%), followed by “Genetic Information Processing” (14.10% ± 0.42%), “Environmental Information Processing” (8.66% ± 0.53%), “Cellular Processes” (8.45% ± 0.12%), “Human Diseases” (6.99% ± 0.10%), and “Organismal Systems” (3.77% ± 0.10%). At KEGG Level 2, 47 functional categories were identified, with pathways showing relative abundance greater than 1% summarized in Supplementary Table 3.

**FIGURE 4:**
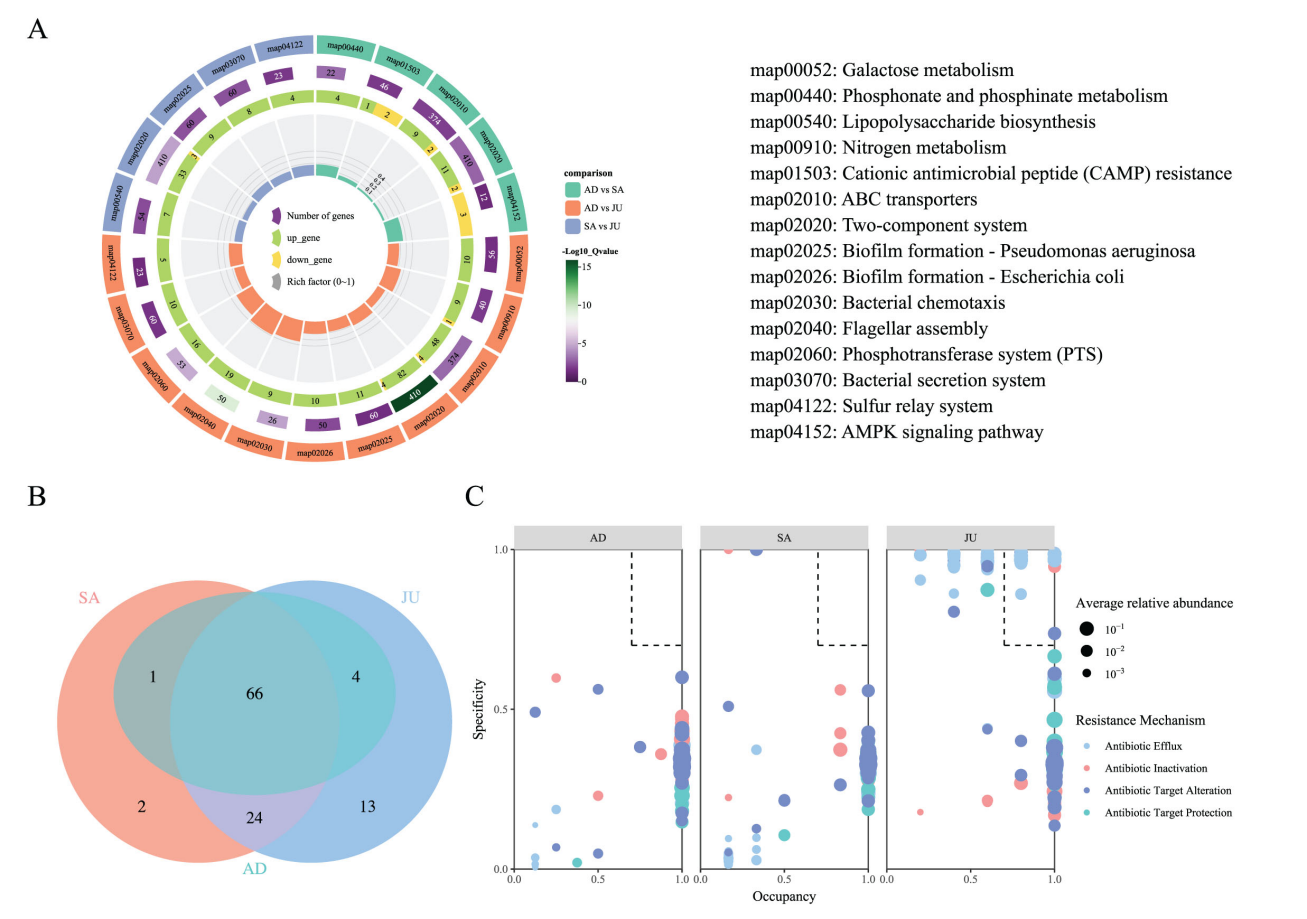
KEGG pathway enrichment and ARO profiles across age groups. **(A)** Multi-layered visualization of KEGG pathway enrichment among age groups. Differentially enriched KEGG level 3 pathways are displayed for each pairwise comparison (AD vs. SA, AD vs. JU, SA vs. JU). From outer to inner rings: pathway IDs (with full names on the right), −log_10_(Q value) and gene count, numbers of upregulated (green) and downregulated (yellow) KOs, and the proportion of differentially expressed genes (upregulated + downregulated) within each enriched pathway, represented by the sector height. Color codes in the outermost ring indicate group comparisons. **(B)** Venn diagram illustrating shared and unique AROs among the three groups. **(C)** SPEC-OCCU plot of AROs. Dot size reflects average relative abundance; color indicates resistance mechanism. Dashed lines at 0.7 mark specificity and occupancy thresholds. AROs within the dashed box meet both criteria.

In this study, KEGG level 3 pathway enrichment analysis was conducted to explore functional differences in the gut microbiota of Mongolian wild asses across different age groups. The results showed that, in the comparison between the AD and SA, pathways such as ABC transporters and the Two-component system were predominantly upregulated, suggesting more active functions related to substrate transport and signal transduction in the SA. In contrast, the AMPK signaling pathway, associated with metabolic regulation, was mainly downregulated, indicating notable functional differences in microbial activity between these two groups (Figure 4A). In the comparison between the AD and JU, multiple KEGG pathways were found to be enriched with differentially expressed KOs. Among them, pathways such as Galactose metabolism and Biofilm formation—Pseudomonas aeruginosa included only KOs upregulated in the JU, suggesting higher functional activity in these pathways at the juvenile stage. Notably, pathways such as Nitrogen metabolism, ABC transporters, and the Two-component system contained both upregulated and downregulated KOs, indicating that these functions may be subject to more complex regulatory patterns between groups. In the comparison between the SA and JU, a total of 410 KOs were annotated in the Two-component system pathway, among which 33 were upregulated and 3 were downregulated in the JU, suggesting heightened activity of this pathway in juveniles. It is important to note that in this study, “upregulated” refers to KOs with higher expression in the latter group of the comparison, while “downregulated” refers to those with lower expression.

#### 3.4.2 CARD analysis

Using the CARD, metagenomic sequencing data were annotated, resulting in the identification of 12,664 UniGenes corresponding to 110 AROs. These AROs were classified into five resistance mechanisms: antibiotic efflux (40.52%), antibiotic inactivation (14.66%), antibiotic target alteration (33.62%), antibiotic target protection (9.48%), and reduced permeability to antibiotic (1.72%). The numbers of detected AROs in the AD, SA, and JU were 52, 57, and 82, respectively, with the JU exhibiting a significantly higher number of AROs than the AD (*p* = 0.0121). Venn diagram analysis (Figure 4B) showed 66 AROs were shared among the three groups; SA and JU shared 24 AROs, while JU had 13 unique AROs. The AD shared only 1 ARO with SA and 4 with JU, without any unique AROs. Notably, the top 30 AROs by relative abundance were shared across all groups and had relative abundances above 0.1%. In contrast, group-specific AROs exhibited relatively low abundance, all below 0.1%.

Using the SPEC-OCCU method, resistance genes with both high specificity (Specificity ≥ 0.7) and broad distribution (Occupancy ≥ 0.7) were selected to identify potential key AROs within the groups (Figure 4C). Seventeen AROs in the JU met these criteria. Among them, 13 belonged to the antibiotic efflux mechanism: AcrE (0.9714, 0.8), AcrF (0.9676, 0.8), TolC (0.9903, 0.8), YojI (0.9596, 0.8), acrB (0.9703, 1), acrD (0.9659, 1), emrK (1.0000, 0.8), evgS (0.9803, 0.8), mdtE (0.9568, 0.8), mdtF (0.9857, 1), mdtG (1.0000, 0.8), mdtO (0.9684, 1), and qacJ (0.8605, 0.8). Three belonged to antibiotic target alteration: Escherichia coli GlpT with mutation conferring resistance to Fosfomycin (0.987714, 1), eptA (0.989296, 0.8), and vanTr gene in vanL cluster (0.737244, 1). One gene, EC-14 (0.946846, 1), was classified under antibiotic inactivation.

#### 3.4.3 CAZy analysis

**FIGURE 5:**
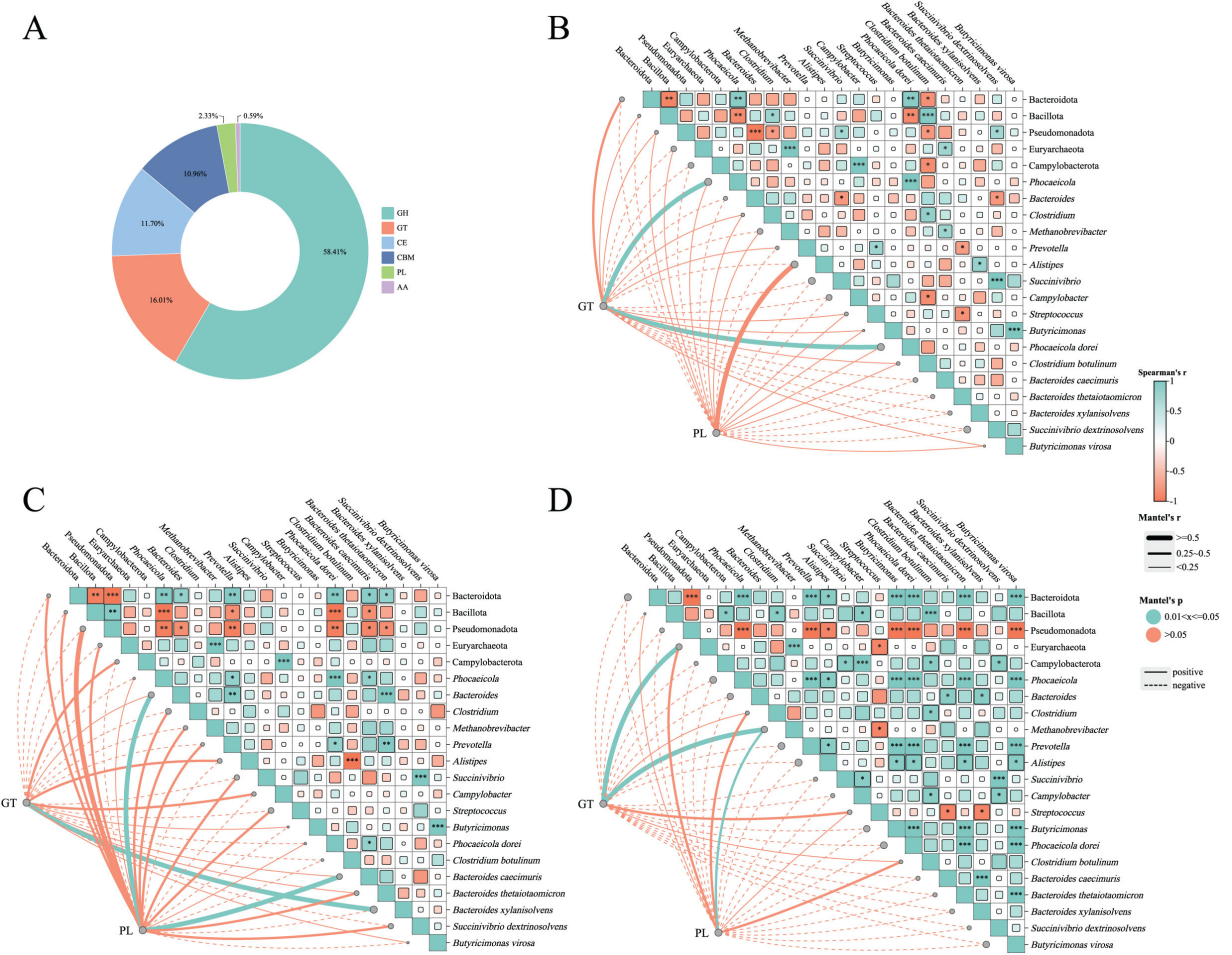
CAZy enzyme composition and correlations with microbial taxa. **(A)** Relative abundance of six CAZy enzyme families in the gut microbiome of Mongolian wild asses: glycoside hydrolases (GH), glycosyltransferases (GT), carbohydrate esterases (CE), carbohydrate-binding modules (CBM), polysaccharide lyases (PL), and auxiliary activities (AA). **(B)** Mantel test and Spearman correlation between GT abundance and microbial taxa in AD. **(C)** Mantel and Spearman correlations between GT or PL abundances and microbial taxa in SA. **(D)** Mantel and Spearman correlations between GT or PL abundances and microbial taxa in JU. **(B–D)** In the heatmap cells, color gradient represents the Spearman’s correlation coefficient (*r*). Asterisks within the cells denote the significance level of Spearman’s *p*-value (*p < 0.05; **p < 0.01, ***p < 0.001). The proportion of colored area within each square reflects the strength of Spearman’s correlation significance. Overlaid line segments represent Mantel’s *r* values, with line thickness indicating effect size. Line color indicates Mantel’s *p*-value significance: pink lines (*p* > 0.05), green lines (0.01 < *p* ≤ 0.05). Solid lines indicate positive correlations, and dashed lines indicate negative correlations.

Based on similarities in amino acid sequences of protein domains, CAZymes can be categorized into six major families: glycoside hydrolases (GH), glycosyltransferases (GT), polysaccharide lyases (PL), carbohydrate esterases (CE), carbohydrate-binding modules (CBM), and auxiliary activities (AA). In this study, GH accounted for the highest proportion (58.41%), followed by GT, CE, CBM, PL, and AA (Figure 5A).

Kruskal–Wallis tests revealed significant differences in GT (*p* = 0.0137) and PL (*p* = 0.0402) among the three groups, with GT levels increasing from AD to SA to JU, while PL levels decreased accordingly. Mantel tests were conducted to assess correlations between GT or PL abundance and microbial taxa at phylum, genus, and species levels. In the AD, GT showed significant positive correlations with *Phocaeicola* and *Phocaeicola dorei* (Figure 5B). In the SA, GT correlated positively with *Bacteroides xylanisolvens*, while PL correlated with *Bacteroides* and *Bacteroides caecimuris* (Figure 5C). In the JU, GT correlated positively with Euryarchaeota and *Methanobrevibacter*, while PL also showed a significant correlation with *Methanobrevibacter* (Figure 5D).

## 4 Discussion

### 4.1 Age-dependent microbial shifts and ecological adaptation

This study found that Bacteroidota and Bacillota are the core bacterial phyla in the gut of Mongolian wild asses, together accounting for more than 85% of relative abundance. A similar gut microbial composition has been observed in closely related species such as the Tibetan wild ass (*Equus kiang*) (Gao et al., 2020), domestic donkey (*Equus asinus*) (Liu et al., 2020), and horse (*Equus caballus*) (Mshelia et al., 2018), suggesting that these two phyla play a universally important role in maintaining gut microbial homeostasis and promoting host health in equids (Sears, 2005; Shang et al., 2016). The observed phylum-level changes across developmental stages suggest that gut microbial communities undergo substantial remodeling as the host matures. Similar patterns have been observed in other wild animals—for instance, in Antarctic chinstrap penguins (*Pygoscelis antarctica*), Bacillota dominate in chicks, while Bacteroidota dominate in adults (Barbosa et al., 2016); in Bengal tigers (*Panthera tigris tigris*), Bacteroidota are more abundant in adults than in subadults (Su et al., 2021). These trends are consistent with those seen across different ontogenetic stages of Mongolian wild asses.

It has been previously reported that, during early mammalian development, the gut microbiota are primarily established through maternal microbial transmission, with Bacillota typically dominating. These bacteria possess strong capabilities for lactose and protein metabolism (Walters et al., 2023), which aids in rapid energy acquisition, maintenance of gut barrier function, and development of initial immune tolerance. This is particularly valuable for Mongolian wild ass juveniles growing in harsh, arid environments. In contrast, Bacteroidota are primarily involved in the degradation of polysaccharides and cellulose, breaking down complex carbohydrates (Ye et al., 2021). However, due to limited intake of fibrous substrates during the juvenile stage, Bacteroidota abundance remains low. As weaning and dietary changes occur, their abundance increases, consistent with prior studies on microbial succession during weaning (Flores et al., 2024). Similar patterns have been observed in the rumen of Tibetan sheep, where the ruminal abundance of Bacteroidota increased from 18.9% at birth to 53.9% at one year of age (Wang et al., 2019). This highlights the significant influence of dietary structure on rumen microbial composition.

Additionally, the ratio of Bacteroidota to Bacillota (B/B ratio) reflects host energy metabolism strategies and environmental adaptability (Palau-Rodriguez et al., 2015; Qin et al., 2025). A high B/B ratio is often associated with increased energy intake and storage, typical during periods of abundant forage (e.g., the breeding season), while a low B/B ratio correlates with enhanced fiber degradation, suitable for cold, arid seasons when coarse fiber becomes the primary energy source (Song et al., 2023). In this study, juveniles exhibited a significantly higher B/B ratio than subadults and adults, indicating a Bacillota-dominated “high-energy” microbial structure that supports rapid development through milk and protein-rich plant intake. This energy-acquisition mode is critical for survival in arid desert ecosystems, enabling juveniles to quickly build necessary energy reserves. As the animals age, dietary diversity and fibrous plant intake increase, Bacteroidota abundance rises accordingly, microbial diversity expands, and the B/B ratio gradually decreases and stabilizes—signifying the establishment of a mature microbial metabolic state tailored to adult dependence on high-fiber, low-nutrient vegetation, thereby enhancing survival adaptability in arid environments.

According to our findings, the relative abundance of Pseudomonadota (formerly Proteobacteria) was significantly higher in the JU than in the AD and SA. LEfSe analysis (LDA value > 4) also identified it as a key biomarker for the juvenile stage. This trend mirrors observations in Tibetan sheep, where Pseudomonadota abundance declined from 40.8% at birth to 5.9% at one year (Wang et al., 2019); in camels, abundance decreased from 17.28% at 2 months to 2.39% and 3.22% at one and three years, respectively (He et al., 2019). In early ontogenetic stages of newborns—including humans and other mammals—residual oxygen in the gut allows Pseudomonadota to act as early colonizers, consuming oxygen and lowering the redox potential, thus facilitating the establishment of strictly anaerobic communities (Shin et al., 2015; Moon et al., 2018). Moreover, Pseudomonadota possess broad substrate utilization capabilities, enabling them to metabolize proteins, carbohydrates, and lipids. Though typically less abundant in adult guts, studies in humans have shown that they play important roles in microbial functional diversity (Bradley and Pollard, 2017). In Mongolian wild ass juveniles, they may help compensate for early dietary limitations and enhance gut functional adaptability (Moon et al., 2018)—a trait especially crucial in arid desert environments where food availability and nutrition are highly variable. Enterobacterales, also identified as a key biomarker in juveniles via LEfSe analysis, are among the first colonizers of the newborn gut. These bacteria utilize rapidly available carbon sources such as glucose and lactose and promote the establishment of anaerobic taxa (e.g., Bacteroidota) by consuming oxygen (Flores et al., 2024).

Concurrently, the genera *Bacteroides*, *Prevotella*, and *Alistipes* showed increasing abundance from JU to SA to AD, with *Prevotella* and *Alistipes* significantly higher in AD and SA compared to JU, all belonging to the phylum Bacteroidota. *Bacteroides* are involved in bile acid, protein, and fat metabolism and can regulate carbohydrate metabolism, while *Alistipes* are associated with short-chain fatty acid metabolism. Both genera are bile-tolerant microorganisms (David et al., 2014) that enhance lipid metabolism through acetate production (Yin et al., 2018). Additionally, *Bacteroides* can modulate T cell differentiation by secreting polysaccharide A, suppressing inflammation and enhancing host immunity (Telesford et al., 2015; Zeng et al., 2019) while *Alistipes* contribute to host immune responses through butyrate and other short-chain fatty acids (Borton et al., 2017). Diets rich in carbohydrates typically favor a *Prevotella*-dominated microbiota (De Filippo et al., 2010), suggesting that after weaning, Mongolian wild asses promote the colonization and functional transformation of dominant taxa such as Bacteroidota by increasing the intake of fibrous, high-carbohydrate plants. This supports enhanced energy acquisition and immune regulation, providing crucial microecological adaptation for survival in an environment with fibrous but nutrient-poor vegetation.

### 4.2 Enhanced diversity and community stability in arid environments

This study further revealed the dynamic changes in gut microbial community structure across different developmental stages of wild Mongolian wild asses by examining both α-diversity and β-diversity. The results showed that Shannon and Simpson indices were significantly higher in the AD and SA compared to the JU, indicating that gut microbial diversity and evenness progressively increase with individual development. This trend is consistent with findings in chinstrap penguins (*Pygoscelis antarctica*), where α-diversity in adults was significantly higher than in chicks (Barbosa et al., 2016). Similarly, in Chongming white goat (*Capra hircus*) kids, gut microbial α-diversity increased markedly with weaning and aging (Liao et al., 2021). Research has shown that the shift from low diversity in juveniles to adult-level diversity is mainly attributed to the introduction of solid food, which provides a rich array of substrates for microbial growth (Flores et al., 2024). Previous studies have indicated that higher α-diversity corresponds to a more complex and stable gut microbiota composition, which is more resistant to disturbances and better adapted to environmental changes, and is closely associated with host health (Shade et al., 2012; Sommer et al., 2017). In the juvenile stage, due to an immature immune system, limited dietary diversity, and maternal transmission of microbes, the gut microbiota remains in a formative and fluctuating state with relatively low diversity. However, with weaning and dietary transitions, food sources become more varied and fiber-rich, exposing the gut microbiota to more exogenous environmental and nutritional factors, leading to increased complexity and stability. This is particularly important in arid desert environments, where plant resources are sparse and variable—higher α-diversity grants the host greater metabolic flexibility and microbial resilience, which helps buffer fluctuations in food composition and environmental pressures.

In addition, NMDS analysis based on Bray–Curtis distances showed that sample points in the AD and SA were more clustered and clearly separated into two groups, while the JU exhibited more dispersed distribution. This suggests greater inter-individual variability in gut microbiota composition during the juvenile stage (JU), where microbial communities are less stable and more strongly influenced by maternal origin, early environmental exposure, and individual developmental differences. As age increases and diet becomes more diverse, the microbial communities in the SA and AD become more stable and mature, with reduced structural variability and enhanced stability and consistency. Overall, the synergistic changes in α-diversity and β-diversity illustrate a clear evolutionary trajectory of the gut microbiota in Mongolian wild asses, from a “low-diversity, low-stability” state in early life to a mature state characterized by “high diversity and distinct structural differentiation.” This enhancement of diversity and refinement of community structure not only strengthens metabolic functions and immune defense but may also provide crucial microbial support for maintaining host health in the harsh arid desert ecosystem.

### 4.3 Functional development of the gut microbiome facilitates adaptation to arid environments

Functional gene analysis is a critical component of gut microbiota research and an essential approach to exploring how microbes regulate host metabolism (Ogata et al., 1999). In this study, KEGG, CARD, and CAZy functional annotations were performed on the gut microbiota of Mongolian wild asses. KEGG annotation results indicated that metabolism-related functions were dominant in the gut microbiota, accounting for 58.03% of the relative abundance. This indicates that gut microbes play a pivotal role in the digestion, absorption, and metabolism of nutrients in the host (Flint et al., 2012), a metabolic capacity that is especially crucial for maintaining energy balance in the resource-scarce and nutritionally volatile desert ecosystems inhabited by Mongolian wild asses. Furthermore, the study revealed significant differences in functional expression among age groups, with pathways such as ABC transporters, Two-component systems, and Nitrogen metabolism exhibiting higher activity in juvenile and subadult stages. This suggests that these functional modules are important for nutritional adaptation and environmental signal response during early development. Similar functional shifts have been observed in the human infant gut microbiome, with studies reporting dynamic changes in carbohydrate utilization, vitamin metabolism, and nitrogen cycling during infancy (Yatsunenko et al., 2012). Germ-free mouse models also confirm that microbial utilization of host-derived carbohydrates is critical for early microbiota establishment (Hooper et al., 1999; Martens et al., 2008). A comparison of functional pathways between the AD and JU showed significant upregulation of galactose metabolism in the JU, likely due to milk being the primary nutrient source during early life. The lactose breakdown product galactose serves as an energy source for microbes via the galactose metabolism pathway, promoting the proliferation of beneficial bacteria and supporting the establishment of a stable gut microbiome. This is consistent with the presence of genes for metabolizing milk- and mucosa-associated carbohydrates in the infant human gut microbiome (Yatsunenko et al., 2012).

The gut, as a complex ecosystem, is closely linked to host immune function and inflammatory responses (Adak and Khan, 2019). Existing research has shown that the gut microbiota plays a crucial role in maintaining health and supporting growth and development in equine species (Costa et al., 2015; Lindenberg et al., 2019). In particular, during the early stages of mammalian development, gut immune protection heavily relies on the transfer of maternal antibodies, including IgA, IgG, and IgM, which are typically supplied via colostrum and milk (Hurley and Theil, 2011). In this study, analysis of the distribution characteristics of AROs showed that the JU had a significantly higher number of AROs than the AD. Moreover, the JU and SA shared a considerable number of AROs, and both contained a relatively rich set of unique AROs. Further analysis using the SPEC-OCCU method identified 17 AROs in the JU that could be classified as potential key resistance genes. These results suggest a potentially higher risk of antibiotic resistance transmission in juvenile Mongolian wild asses, or they may reflect that the early-stage microbiota is still in a formative and fluctuating phase, influenced by maternal antibodies, environmental microbial pressure, and changes in diet. In arid desert environments, where external microbial pressures are intense, the dynamic changes in juvenile gut microbiota and their resistance genes may play an important role in shaping the host’s early immune defenses and in adapting to complex ecological factors.

Carbohydrates serve as the primary material basis for host energy supply and life metabolism. Their metabolic processes are crucial not only for energy production, growth, and substance transformation in the host, but also as a vital energy source for gut microbiota (Tian et al., 2021). In arid desert habitats, plants typically exhibit high fiber content, low moisture, high lignin, and strong stress resistance. These structurally complex and hard-to-digest plants place high demands on microbial degradation capabilities. Therefore, the ability of wild animals to digest and absorb desert plants is critical for their energy acquisition and adaptation to desert environments. Results of this study showed that the gut microbiota of Mongolian wild asses was dominated by glycoside hydrolases (GHs) and glycosyltransferases (GTs), accounting for 58.41% and 16.01% of the total CAZyme abundance, respectively. This distribution is broadly consistent with that observed in the gut microbiota of other mammals, where these enzymes are mainly involved in the degradation of plant polysaccharides such as cellulose and hemicellulose (Kaoutari et al., 2013; Gong et al., 2020). The abundance of GTs and polysaccharide lyases (PLs) showed distinct functional shifts across developmental stages. GTs were more abundant in the JU, possibly reflecting microbial functional adaptation to the milk-feeding environment. While GTs do not directly degrade milk carbohydrates, they play a synergistic role in the milk oligosaccharide metabolic network by synthesizing extracellular polysaccharides (EPS) and glycoproteins, enhancing microbial adhesion and colonization in the gut, promoting microbial cooperation, and improving the metabolic efficiency of milk components. The abundance of PLs was significantly higher in AD than in the JU, suggesting that with host development, the microbial community’s ability to degrade complex polysaccharides improves. PLs primarily target complex carbon sources such as pectin and polygalacturonic acid in plant cell walls. The enrichment of such enzymes is likely associated with the adult Mongolian wild asses’ diet, which includes a higher proportion of structurally complex hay and fibrous plants. Efficient degradation of these carbon sources allows gut microbes to provide a stable and sustained energy supply for the host. Previous studies have shown that Bacteroidota play a key role in the degradation of complex, indigestible dietary polysaccharides in the mammalian gut (Flint et al., 2012). In this study, Mantel test results also demonstrated significant positive correlations between Bacteroidota-associated microbes and GT and PL enzyme activities in the AD and SA. Moreover, the relative abundance of Bacteroidota increased from 39.53% in the JU to 61.78% and 64.92% in the SA and AD, respectively. This suggests that as the abundance of Bacteroidota increases in the host gut, their role in cellulose and polysaccharide degradation becomes more prominent, further facilitating stable energy supply and enhancing the host’s adaptive capacity to arid desert environments.

In this study, a multi-omics approach was employed to systematically analyze the compositional and functional characteristics of gut microbiota across different developmental stages of Mongolian wild asses, revealing the potential roles of the microbiome in host growth, development, and adaptation to desert ecosystems. However, certain limitations remain. This study has several limitations. First, the sample size was relatively small, especially for juveniles (*n* = 5), which may reduce statistical power. Increasing sample sizes across age classes and geographic regions would improve the robustness and generalizability of the findings. Second, age classification was inferred from fecal size and morphology; future studies should combine pellet morphometrics with infrared camera monitoring, field observations, and, where possible, body measurements or genetic markers to refine classification criteria. Third, only females were included in the final dataset, and incorporating males will be necessary to assess potential sex-related differences. Finally, as a cross-sectional study, our design does not capture longitudinal changes; future long-term monitoring will be crucial to reveal the temporal succession and ecological significance of gut microbiota across developmental stages. Overall, this study lays a foundation for understanding the gut microecological functions of wild equids. Future research that integrates higher-resolution techniques and interdisciplinary approaches will facilitate a more comprehensive understanding of the complex relationships between gut microbiota, host health, and adaptation to harsh environments such as arid deserts, thereby offering theoretical support for the scientific conservation of endangered wild animals such as the Mongolian wild ass.

## Data Availability

The datasets presented in this study are publicly available. The raw sequence data reported in this paper have been deposited in the Genome Sequence Archive (Genomics, Proteomics & Bioinformatics 2021) in National Genomics Data Center (Nucleic Acids Res 2022), China National Center for Bioinformation / Beijing Institute of Genomics, Chinese Academy of Sciences (GSA: CRA029385), and are accessible at https://ngdc.cncb.ac.cn/gsa.
