# Supplementary material for: Developmental dynamics and functional adaptation of gut microbiota in Mongolian wild asses (Equus hemionus hemionus) across ontogenetic stages in arid desert ecosystems
Source: Front Microbiol. 2025 Sep 17;16:1659661. doi: 10.3389/fmicb.2025.1659661 (PMC12486412; doi:10.3389/fmicb.2025.1659661)
Supplement: Supplementary file 1 [file Supplementary_file_1.docx]

**
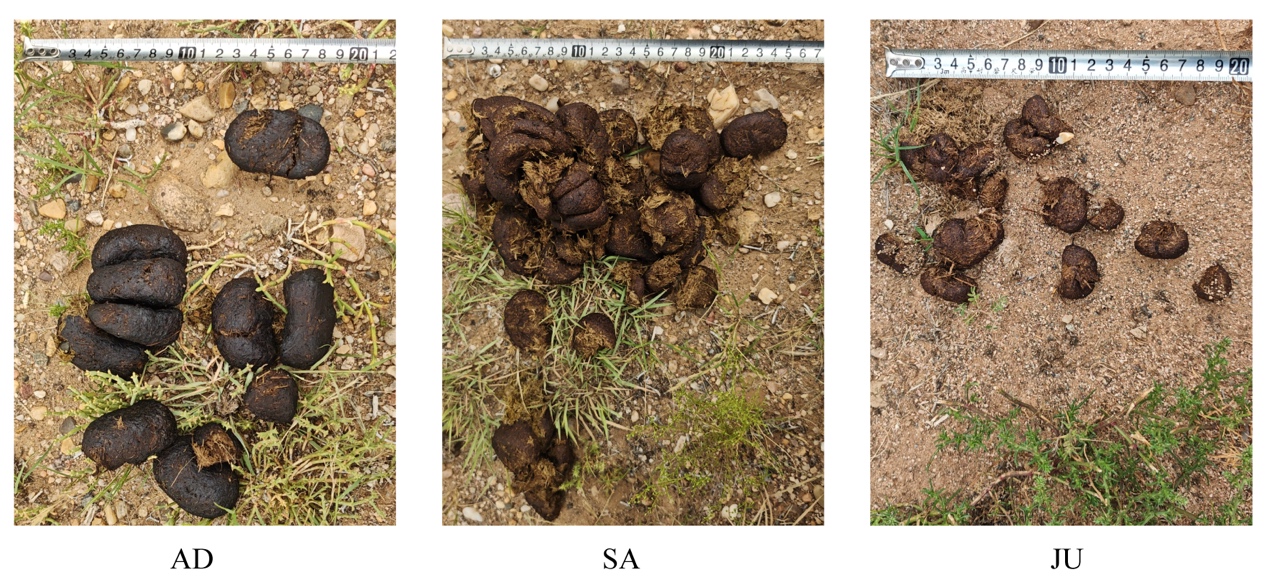
**

**Fig. S1** Representative fecal samples of adult (AD), subadult (SA), and juvenile (JU) Mongolian wild asses. The size of fecal pellets is shown with a measuring tape for scale.

**Table S1 α-diversity indices of gut microbiota at the species level.**

| **Sample** | **Observed_species** | **Chao1** | **Shannon** | **Simpson** | **PD_whole_tree** |
| --- | --- | --- | --- | --- | --- |
| AD11-1 | 108 | 157.043478 | 4.81608838 | 0.93442044 | 57.65573 |
| AD11-2 | 85 | 136.230769 | 4.7241043 | 0.94116284 | 50.2747 |
| AD11-3 | 95 | 113.125 | 4.8429869 | 0.93969753 | 54.66864 |
| AD11-4 | 95 | 134.176471 | 4.71554516 | 0.93478676 | 53.77105 |
| AD11-5 | 92 | 123.5 | 4.72184704 | 0.93593327 | 51.83338 |
| AD11-6 | 90 | 142 | 4.52548343 | 0.92344249 | 52.50518 |
| AD11-7 | 99 | 134.052632 | 4.71494261 | 0.93102448 | 54.7731 |
| AD11-8 | 105 | 177.769231 | 4.80301211 | 0.92703646 | 59.57862 |
| SA11-1 | 109 | 171.666667 | 4.93917735 | 0.93935299 | 59.81753 |
| SA11-2 | 94 | 153.125 | 4.611679 | 0.9260147 | 52.26026 |
| SA11-3 | 98 | 119.12 | 4.91852185 | 0.94231133 | 54.68514 |
| SA11-4 | 106 | 143.272727 | 4.90532538 | 0.9333967 | 56.14194 |
| SA11-5 | 104 | 150.3125 | 4.56535788 | 0.88508898 | 55.84648 |
| SA11-6 | 97 | 155.571429 | 4.66177559 | 0.92715725 | 53.46951 |
| JU11-1 | 100 | 202.083333 | 4.64893811 | 0.92472959 | 56.91963 |
| JU11-2 | 73 | 100 | 4.38664839 | 0.924888 | 44.3425 |
| JU11-3 | 83 | 116.157895 | 3.74976485 | 0.7943843 | 48.70161 |
| JU11-4 | 91 | 214.75 | 3.83363154 | 0.79019826 | 53.27757 |
| JU11-5 | 82 | 127.769231 | 4.41381745 | 0.92216727 | 48.00326 |

**Table S2 Taxonomic composition and statistical comparisons of microbial communities at the phylum, genus, and species levels derived from fecal samples of Mongolian wild asses.**

| **Taxonomic rank** | **Taxon Name** | **AD Mean(%)** | **AD SD** | **SA Mean(%)** | **SA SD** | **JU Mean(%)** | **JU SD** | **Kruskal-Wallis adj.p** | **AD vs SA (adj.p)** | **AD vs JU (adj.p)** | **SA vs JU (adj.p)** |
| --- | --- | --- | --- | --- | --- | --- | --- | --- | --- | --- | --- |
| Phylum | Bacteroidota | 64.923204 | 3.225208 | 61.776619 | 8.928652 | 39.534375 | 16.894499 | 0.055959 |  |  |  |
|  | Bacillota | 27.047578 | 4.578011 | 28.601718 | 6.503828 | 30.214318 | 14.821201 | 0.959400 |  |  |  |
|  | Pseudomonadota | 0.954418 | 0.527642 | 2.795251 | 3.115972 | 26.074598 | 28.298520 | 0.023354 | 0.282384 | 0.004662 | 0.045455 |
|  | Euryarchaeota | 4.963756 | 1.440013 | 4.559668 | 1.983409 | 1.794686 | 0.626783 | 0.023354 | 0.572761 | 0.004662 | 0.006494 |
|  | Campylobacterota | 1.291115 | 2.215722 | 1.285998 | 1.376550 | 1.726359 | 1.792325 | 0.384736 |  |  |  |
| Genus | *Phocaeicola* | 36.381192 | 3.691175 | 33.657562 | 5.003230 | 23.027630 | 9.935735 | 0.072078 |  |  |  |
|  | *Bacteroides* | 9.612022 | 0.561342 | 9.588423 | 1.142283 | 6.748302 | 3.192050 | 0.237499 |  |  |  |
|  | *Clostridium* | 4.562685 | 4.802038 | 2.572920 | 2.634567 | 13.126706 | 16.756265 | 0.781900 |  |  |  |
|  | *Methanobrevibacter* | 4.797847 | 1.416137 | 4.384724 | 1.880000 | 1.729386 | 0.618252 | 0.023354 | 0.572761 | 0.004662 | 0.006494 |
|  | *Prevotella* | 2.833015 | 0.426568 | 2.660168 | 0.458752 | 1.368731 | 0.643992 | 0.023354 | 0.413586 | 0.009324 | 0.012987 |
|  | *Alistipes* | 2.175162 | 0.300920 | 1.918090 | 0.617201 | 0.954481 | 0.314444 | 0.023354 | 0.413586 | 0.004662 | 0.012987 |
|  | *Succinivibrio* | 0.680186 | 0.332027 | 1.358623 | 1.777212 | 3.676100 | 5.190505 | 0.959400 |  |  |  |
|  | *Campylobacter* | 1.291115 | 2.215722 | 1.285998 | 1.376550 | 1.726359 | 1.792325 | 0.384736 |  |  |  |
|  | *Streptococcus* | 1.388302 | 0.977650 | 0.899007 | 0.776147 | 1.276468 | 1.152513 | 0.437089 |  |  |  |
|  | *Butyricimonas* | 1.158537 | 0.313381 | 1.513272 | 0.503974 | 0.377102 | 0.188086 | 0.023354 | 0.181152 | 0.004662 | 0.006494 |
| Species | *Phocaeicola dorei* | 35.228153 | 3.644184 | 32.462746 | 4.976643 | 22.177126 | 9.550017 | 0.072078 |  |  |  |
|  | *Clostridium botulinum* | 4.073825 | 4.817769 | 0.147857 | 0.116674 | 12.543581 | 16.934530 | 0.237499 |  |  |  |
|  | *Bacteroides caecimuris* | 4.454265 | 0.655177 | 4.558458 | 0.562488 | 3.540744 | 1.673170 | 0.321665 |  |  |  |
|  | *Bacteroides thetaiotaomicron* | 2.383310 | 0.401682 | 2.089405 | 0.733189 | 1.071687 | 0.655930 | 0.058472 |  |  |  |
|  | *Bacteroides xylanisolvens* | 1.792023 | 0.290184 | 1.899106 | 0.243051 | 1.405768 | 0.499513 | 0.285605 |  |  |  |
|  | *Succinivibrio dextrinosolvens* | 0.680186 | 0.332027 | 1.358623 | 1.777212 | 3.676100 | 5.190505 | 0.959400 |  |  |  |
|  | *Butyricimonas virosa* | 1.156374 | 0.314058 | 1.508727 | 0.501530 | 0.376462 | 0.187856 | 0.023354 | 0.181152 | 0.004662 | 0.006494 |

**Table S3 Relative abundance of KEGG Level 2 pathways (>1%) in the AD, SA, and JU**

| **KEGG Level 2 Pathways** | **AD (Mean)** | **AD (SD)** | **SA (Mean)** | **SA (SD)** | **JU (Mean)** | **JU (SD)** |
| --- | --- | --- | --- | --- | --- | --- |
| Carbohydrate metabolism | 14.575628 | 0.082805 | 14.566781 | 0.124728 | 14.461970 | 0.075087 |
| Amino acid metabolism | 8.340794 | 0.035010 | 8.263993 | 0.068359 | 8.225241 | 0.063987 |
| Glycan biosynthesis and metabolism | 6.839088 | 0.093622 | 6.881811 | 0.050364 | 7.027257 | 0.109771 |
| Energy metabolism | 5.831484 | 0.061455 | 5.724709 | 0.121231 | 5.556905 | 0.141033 |
| Metabolism of cofactors and vitamins | 5.550923 | 0.037341 | 5.540641 | 0.037532 | 5.655918 | 0.039097 |
| Replication and repair | 5.385211 | 0.061188 | 5.337320 | 0.072005 | 5.320702 | 0.138295 |
| Translation | 5.121402 | 0.104456 | 4.968678 | 0.200524 | 4.630743 | 0.228253 |
| Nucleotide metabolism | 4.680284 | 0.030193 | 4.673813 | 0.020020 | 4.636920 | 0.036278 |
| Membrane transport | 4.248685 | 0.102314 | 4.420991 | 0.237732 | 4.504016 | 0.296626 |
| Signal transduction | 4.054590 | 0.049169 | 4.316119 | 0.343834 | 4.580946 | 0.309383 |
| Lipid metabolism | 4.073442 | 0.032790 | 4.058826 | 0.043375 | 4.219226 | 0.058001 |
| Cellular community - prokaryotes | 3.794223 | 0.028535 | 3.856357 | 0.083669 | 3.873721 | 0.068452 |
| Folding, sorting and degradation | 2.982059 | 0.015919 | 2.946468 | 0.059736 | 2.892775 | 0.075420 |
| Biosynthesis of other secondary metabolites | 2.854069 | 0.032594 | 2.814806 | 0.070205 | 2.766961 | 0.038455 |
| Metabolism of other amino acids | 2.836530 | 0.029828 | 2.826154 | 0.055965 | 2.748954 | 0.030363 |
| Drug resistance: antimicrobial | 2.465900 | 0.016232 | 2.518022 | 0.089451 | 2.635403 | 0.114159 |
| Cell growth and death | 2.261210 | 0.034047 | 2.228574 | 0.048531 | 2.203500 | 0.095555 |
| Transport and catabolism | 1.760373 | 0.065537 | 1.665633 | 0.141956 | 1.702722 | 0.159432 |
| Infectious disease: bacterial | 1.467994 | 0.016911 | 1.478868 | 0.023229 | 1.471682 | 0.022278 |
| Metabolism of terpenoids and polyketides | 1.394876 | 0.009748 | 1.378477 | 0.035027 | 1.365369 | 0.027692 |
| Xenobiotics biodegradation and metabolism | 1.206739 | 0.019453 | 1.220232 | 0.026618 | 1.223013 | 0.034892 |
| Endocrine system | 1.085577 | 0.010174 | 1.086185 | 0.012602 | 1.099879 | 0.016028 |
